# Decrease in brain complexity with methylphenidate treatment in boys diagnosed with attention deficit hyperactivity disorder: An entropy-based qeeg analysis

**DOI:** 10.1192/j.eurpsy.2021.1685

**Published:** 2021-08-13

**Authors:** F.H. Çetin, M.B. Usta, S. Aydın, A.S. Güven

**Affiliations:** 1 Child And Adolescent Psychiatry, Selçuk University, Faculty of Medicine, Konya, Turkey; 2 Child And Adolescent Psychiatry, Ondokuz Mayıs University, Faculty of Medicine, Samsun, Turkey; 3 Biophysics Department, Hacettepe University Medical Faculty, Ankara, Turkey; 4 Pediatric Neurology, Necmettin Erbakan Univesity, Meram Faculty of Medicine, Konya, Turkey

**Keywords:** ADHD, methylphenidate, entropy, complexity

## Abstract

**Introduction:**

Attention deficit hyperactivity (ADHD) disorder is a common childhood neurodevelopmental disorder, and Methylphenidate (MPH) is a first-line therapeutic option for treating ADHD.However, how brain complexity and entropy changes with methylphenidate treatment the clinical implications of possible changes in entropy and the clinical implications of possible changes in entropy have yet to be studied.

**Objectives:**

This study aimed to reveal how the MPH treatment affects the complexity in the brain of children with ADHD by entropy-based qEEG analysis. In addition, the presence of the relationship between possible neurophysiological changes to be detected with clinical variables and how they are two other important questions of this study to be answered.

**Methods:**

During eyes-open resting, EEG signals were recorded from 25 boys with ADHD-combined type before MPH administration and at the end of the 1st month of the treatment. Approximate entropy (ApEn), sample entropy (SampEn), permutation entropy (PermEn) were used to analyse.

**Results:**

**Figure:**
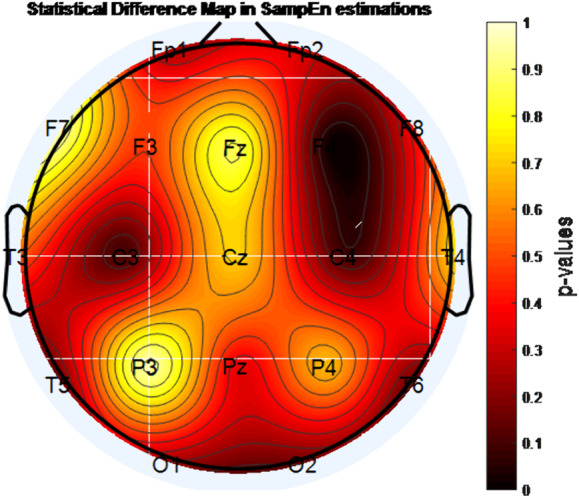

**Figure:**
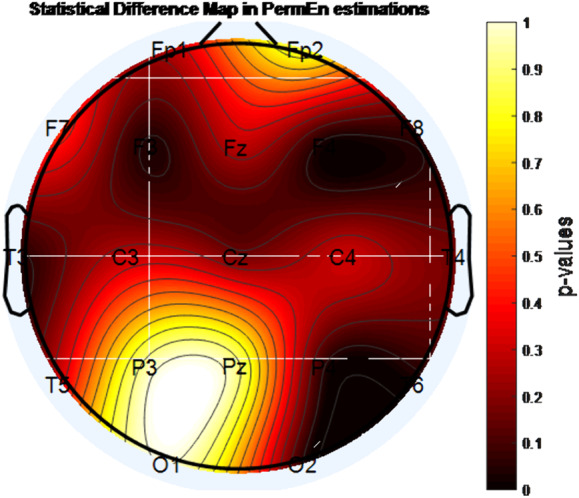

A statistically significant decrease in entropy level was found with MPH treatment in the F4 channel according to approximate entropy (ApEn) and sample entropy (SampEn) analysis (p<0.05). In addition, according to permutation entropy (PermEn) analysis, the decrease in entropy with MPH treatment in the regions indicated by F3, F4, P4, T3, T6, and O2 channels was found to be statistically significant (p <0.05).

**Conclusions:**

This is the first study to investigate how MPH treatment affects the complexity in the brain of children with ADHD. Entropy-based qEEG analysis may be a new method that can be used in diagnostic, clinical and prognostic predictions in ADHD.

**Disclosure:**

No significant relationships.

